# The methyl binding domain 3/nucleosome remodelling and deacetylase complex regulates neural cell fate determination and terminal differentiation in the cerebral cortex

**DOI:** 10.1186/s13064-015-0040-z

**Published:** 2015-05-02

**Authors:** Erin Knock, João Pereira, Patrick D Lombard, Andrew Dimond, Donna Leaford, Frederick J Livesey, Brian Hendrich

**Affiliations:** Wellcome Trust - Medical Research Council Stem Cell Institute, University of Cambridge, Cambridge, CB2 1QR, UK; Gurdon Institute, University of Cambridge, Cambridge, CB2 1QW, UK; Department of Biochemistry, University of Cambridge, Cambridge, CB2 1QN, UK; Tanz Centre for Research in Neurodegenerative Diseases, Krembil Discovery Tower, 6KD-404, 60 Leonard Avenue, Toronto, ON Canada

**Keywords:** Neural progenitors, Neural differentiation, Gene expression

## Abstract

**Background:**

Chromatin-modifying complexes have key roles in regulating various aspects of neural stem cell biology, including self-renewal and neurogenesis. The methyl binding domain 3/nucleosome remodelling and deacetylation (MBD3/NuRD) co-repressor complex facilitates lineage commitment of pluripotent cells in early mouse embryos and is important for stem cell homeostasis in blood and skin, but its function in neurogenesis had not been described. Here, we show for the first time that MBD3/NuRD function is essential for normal neurogenesis in mice.

**Results:**

Deletion of MBD3, a structural component of the NuRD complex, in the developing mouse central nervous system resulted in reduced cortical thickness, defects in the proper specification of cortical projection neuron subtypes and neonatal lethality. These phenotypes are due to alterations in PAX6+ apical progenitor cell outputs, as well as aberrant terminal neuronal differentiation programmes of cortical plate neurons. Normal numbers of PAX6+ apical neural progenitor cells were generated in the MBD3/NuRD-mutant cortex; however, the PAX6+ apical progenitor cells generate EOMES+ basal progenitor cells in reduced numbers. Cortical progenitor cells lacking MBD3/NuRD activity generate neurons that express both deep- and upper-layer markers. Using laser capture microdissection, gene expression profiling and chromatin immunoprecipitation, we provide evidence that MBD3/NuRD functions to control gene expression patterns during neural development.

**Conclusions:**

Our data suggest that although MBD3/NuRD is not required for neural stem cell lineage commitment, it is required to repress inappropriate transcription in both progenitor cells and neurons to facilitate appropriate cell lineage choice and differentiation programmes.

**Electronic supplementary material:**

The online version of this article (doi:10.1186/s13064-015-0040-z) contains supplementary material, which is available to authorized users.

## Background

The production of differentiated neuronal cell types in the developing brain is tightly regulated both spatially and temporally. During the course of cerebral cortex development, neural progenitor cells generate specific subsets of cortical neurons in a characteristic temporal order and then proceed to produce to glial cells [1]. Neural progenitor cells must be able to respond to extracellular signals at the appropriate developmental stage in order to initiate the transcriptional programmes specific to a particular cell lineage. Although the molecular mechanisms underlying specific lineage decisions made by neural progenitors are not fully understood, control of gene expression by chromatin-modifying protein complexes is likely to play a key role in enabling neural precursors to interpret developmental cues instructing them to execute lineage-specific transcriptional programmes (reviewed in [2]).

Apical progenitor cells can divide symmetrically to produce two apical progenitors or asymmetrically to produce one apical progenitor and one neuron or an intermediate/basal progenitor cell [3]. Intermediate or basal progenitor cells are found in the sub-ventricular zone (SVZ) of the developing cortex. These progenitor cells are capable of symmetric division to produce two neurons and function to increase the neurogenic capacity of the apical progenitors [3].

The nucleosome remodeling and deacetylation (NuRD) complex is a multiprotein transcriptional co-repressor complex which has been shown to control cell fate decisions in a variety of developmental contexts in both plants and animals [4,5]. The main enzymatic components of NuRD are provided by the ATPase/nucleosome remodelling component (CHD4, CHD3 or CHD5) and the class I histone deacetylases 1/2 (HDAC1/2). Methyl-CpG binding domain 2 (MBD2) and methyl-CpG binding domain 3 (MBD3) are mutually exclusive in NuRD [6]. However, while MBD2-null mice are viable and fertile, MBD3 is essential for embryonic development [7,8]. There is no evidence that MBD3 exists or functions outside of the NuRD complex. Biochemical purification of Mbd3 from HeLa cells [9,10] and in embryonic stem (ES) cells (BH Lab, PhD Theses and unpublished) confirms that the only significant MBD3 interactions are with NuRD components. MBD3 has been shown to be a key structural protein of NuRD, such that without MBD3, the complex does not form [11,12]. MBD3/NuRD plays an important role in various developmental processes, while MBD2/NuRD (also known as Mecp1) is not essential but rather plays more evolutionarily important roles [13]. In mice, MBD3/NuRD activity is important for developmental transitions of pluripotent cells, as well as for appropriate stem cell homeostasis in haematopoietic and epithelial development [8,12,14-17]. MBD3/NuRD components are expressed in the developing mouse neocortex in areas where neural progenitors reside and are implicated in control of neural identity during neurogenesis [18-20]. Recently, a role for one MBD3/NuRD component protein, CHD4, in promoting synaptic connectivity in postnatal mouse brain has been described [21], but the function of MBD3/NuRD during neurogenesis has not been reported.

In this study, we report the function of the MBD3/NuRD complex during neural development in mice. Conditional deletion of the two main isoforms of MBD3 in the neural lineage revealed that MBD3/NuRD is necessary for PAX6+ apical progenitor cells to implement appropriate lineage decisions. We also find that MBD3/NuRD functions to repress the deep-layer transcriptional programme in postmitotic neurons. In contrast to ES cells, MBD3/NuRD is not required for lineage commitment in neural stem cells but is necessary for neural progenitors to differentiate properly into all cell types of the neocortex.

## Results

### Loss of MBD3/NuRD function results in fewer cortical neurons

**Figure 1:**
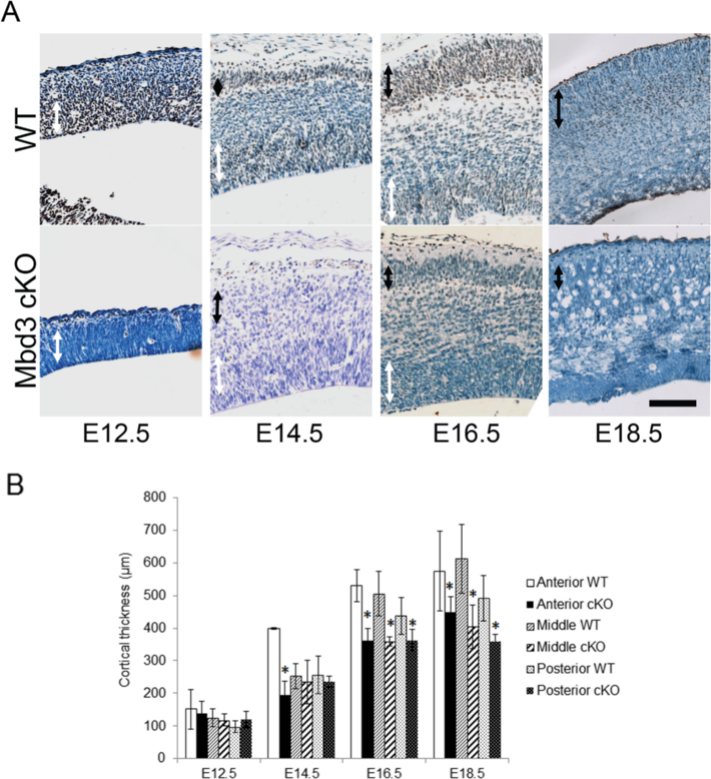
Characterisation of *Mbd3*-mutant brains. **(A)** Representative MBD3 immunostaining (brown) on haematoxylin-stained coronal sections of E12.5, 14.5, 16.5 and 18.5 embryonic wild-type (WT) and mutant (Mbd3 cKO) brains. Black arrows denote the presumptive cortical plate, and white arrows denote the presumptive VZ. Wild-type and mutant sections were stained together on one slide, so the wild type acts as a positive control for staining in the mutant section. Scale bar = 100 μm. **(B)** Mean measurements of cortical thickness from three non-consecutive coronal sections per brain in wild-type (WT) and *Mbd3* cKO) embryos. *N* = 3-6. *E14.5 *P* = 0.0192, *df* = 2; E16.5 *P* = 0.0001, *df* = 2; E18.5 *P* = 0.0001, *df* = 2. Error bars represent st. dev.

MBD3 is expressed in cells of the ventricular zone (VZ) and SVZ at E12.5 and from E14.5 is also expressed in a sub-population of cortical plate neurons (Figure 1A). Embryos in which exon 1 of the *Mbd3* gene had been deleted using the Nestin-Cre transgene (conditional knockout or cKO) showed no anti-MBD3 staining in either of these areas from E12.5 (Figure 1A, Additional file 1: Figure S1B). Nestin-Cre was chosen as this provides expression of Cre from early on in neural development (prior to PAX6 expression) but would not delete Mbd3 in very early embryonic development when Mbd3 is essential [8,22-24]. While Cre-mediated excision of the floxed *Mbd3* allele used in this study results in loss of MBD3A and MBD3B only, no anti-MBD3 reactivity was detectable in the brains of cKO embryos after E12.5, indicating that MBD3C is not significantly expressed in the developing cortex. Nervous system-specific deletion of MBD3 resulted in a significantly smaller cerebral cortex from approximately the mid-point of the cortical neurogenic period (E14.5; Figure 1, Additional file 1: Figure S1C). The size difference was only detected in the anterior sections at E14.5 but was observed in all areas by E16.5 which is consistent with the anterior-posterior gradients of neurogenesis in the mouse cortex. The relative thickness of the MBD3-null cortex was significantly thinner than that of littermate controls throughout development and was approximately 75% of the thickness of littermate controls at E18.5 (Figure 1B).

**Figure 2:**
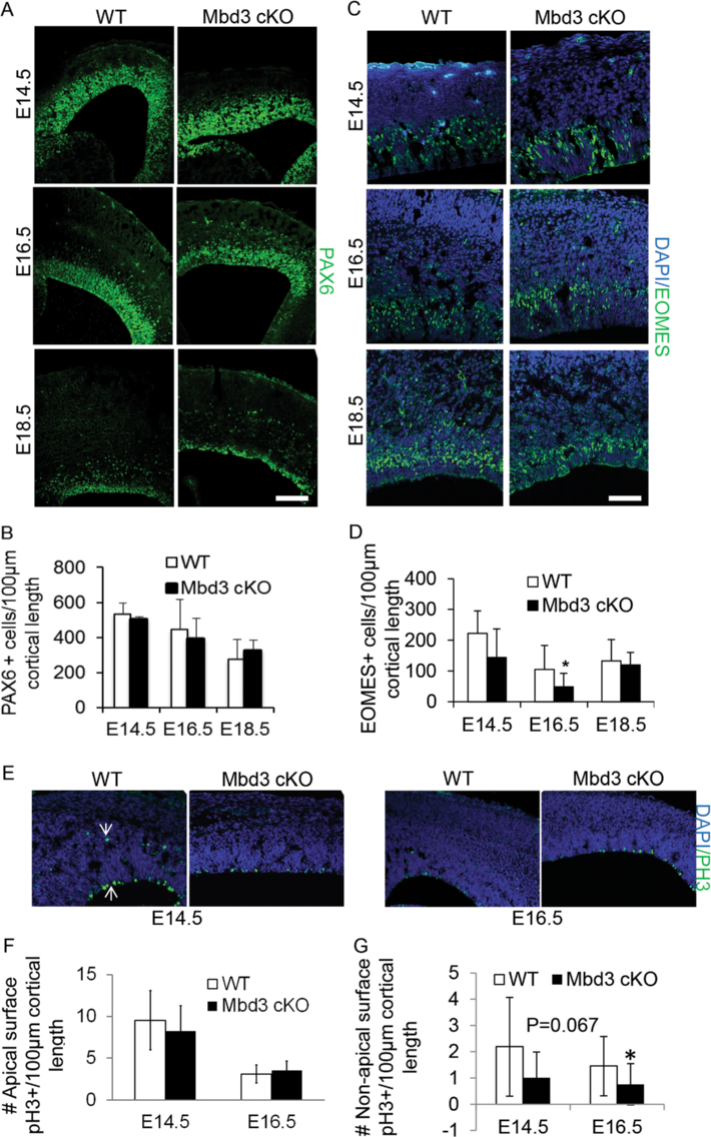
*Mbd3*-deficient embryonic brains show reduced production of EOMES+ basal neural progenitors and reduced neural output. **(A)** Representative immunostaining of E14.5, E16.5 and E18.5 coronal brain sections for PAX6. **(B)** Quantification of stained cells per 100 μm of cortical length. **(C)** Representative immunostaining of E14.5, E16.5 and E18.5 coronal brain sections for EOMES with DAPI counterstain. **(D)** Quantification of stained cells per 100 μm of cortical length. *N* = 3 to 6 *P = 0.0100, *df* = 2. **(E)** Representative immunostaining of E14.5 and E16.5 coronal brain sections for phosphorylated histone H3 (pH3). White arrows indicate the positions of apical and non-apical pH3+ cells. **(F)** Quantification of the number of apical surface pH3+ cells at E14.5 and E16.5 per 100 μm of cortical length, *N* = 3. **(G)** Quantification of the number of non-apical surface pH3+ cells at E14.5 (*P* = 0.067, *df* = 2) and at E16.5, *N* = 3, *P = 0.029, *df* = 2. Error bars represent st. dev. Scale bar = 100 μm.

To determine whether this phenotype resulted from a loss or reduction of any specific cell population, we quantified the number of PAX6+ apical and EOMES+ basal progenitors present in mutant and wild-type (WT) cortices. Quantification of PAX6-expressing apical progenitors in the VZ at E14.5, 16.5 and 18.5 indicated no significant difference between mutant and wild-type cortices at any time point (Figure 2A,B). In contrast, MBD3 cKO cortices had significantly fewer EOMES-expressing basal progenitor cells than WT controls at E16.5 (Figure 2C,D). We also counted the number of phosphorylated histone H3 (pH3)-positive cells at the apical surface of the VZ from three non-consecutive sections of five mice per genotype. We observed no change in the number of cells in mitosis at the apical surface when we compared WT and cKO mice (Figure 2E,F). When we counted the number of non-apical pH3-positive cells from the experiment mentioned above, we observed a borderline significant decrease in the cortex of cKO at E14.5 and a significant decrease at E16.5 compared to WT mice (Figure 2E,G). These data are consistent with mutant brains containing a reduced number of EOMES+ basal progenitors at E16.5. The borderline significant change observed at E14.5 suggests that there may be subtle changes in cell divisions in EOMES+ basal progenitors although we could not detect any change in cell number at this stage.

**Figure 3:**
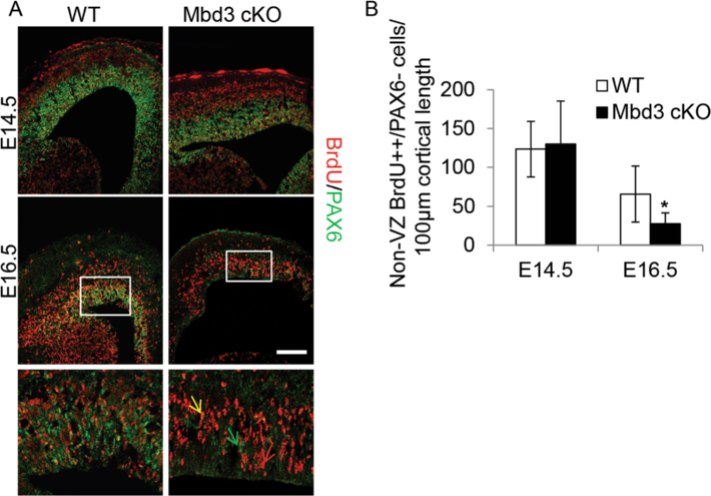
Mbd3cKO mice produce fewer differentiated cells. **(A)** Pregnant mice were injected with a single pulse of BrdU 24 h prior to examination of the embryos at the indicated time points. Differentiated cell output was defined as the number of strongly BrdU (++)/PAX6− cells outside the VZ produced in that 24-h period. Representative immunostaining of E14.5 and E16.5 coronal brain sections for BrdU (red) and PAX6 (green). The white line represents the presumptive boundary between the VZ and outside the VZ (non-VZ). The bottom panels are a magnified image of the boxed area in the above picture. The green, red and yellow arrows point out a PAX6 only +, strong BrdU only + and PAX6/BrdU double-positive cell, respectively. **(B)** Quantification of the number of differentiated neurons produced in 24 h. Scale bar = 100 μm; *N* = 3 to 6, *P = 0.0134, *df* = 2. Error bars represent st. dev. Scale bar = 100 μm.

One role proposed for EOMES-expressing basal progenitor cells is to amplify the neurogenic output of PAX6+ apical progenitors [3]. Therefore, fewer EOMES+ basal progenitors would be predicted to result in reduced neuronal output and could thereby explain the reduction in cortical thickness observed in MBD3-mutant embryos. To test this hypothesis, differentiated cell output was measured over a 24-h period by injecting pregnant dams with a single dose of bromodeoxyuridine (BrdU) at E13.5 or E15.5 and examining the embryos 24 h later. Differentiated cells generated during the BrdU pulse were defined as those cells showing strong staining for BrdU label but not expressing PAX6 and which had migrated outside the VZ and SVZ. This experiment indicated that while cKO embryos produced normal numbers of differentiated cells between E13.5 and E14.5, they show a significantly reduced output of differentiated cells between E15.5 and E16.5 (Figure 3A,B). The differentiated cells in this experiment could be either EOMES+ basal progenitors, neurons, glia or oligodendrocytes. Glia and oligodendrocytes are not produced until much later in development (E18.5 on), so we have excluded these as being the cells we observe. Given that the numbers of neurons produced is much higher than the number of EOMES+ basal progenitors and that we are looking largely in the cortical plate, we (like others [25]) have made the assumption that the majority of cells we are counting are neurons. This finding is consistent with the predicted reduction in neurons generated from the reduced number of EOMES+ basal progenitor cells in the MBD3-null cortex.

### Altered progenitor cell proliferation in MBD3/NuRD-mutant cortices

**Figure 4:**
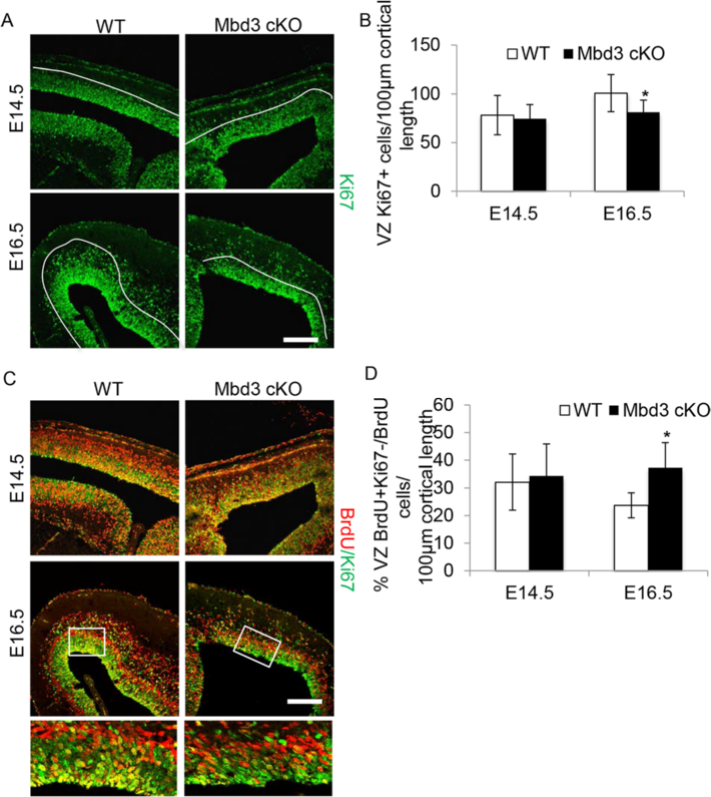
Fewer *Mbd3*-deficient PAX6+ apical progenitors divide between E15.5 and E16.5. **(A)** Representative immunostaining of E14.5 and E16.5 coronal brain sections for Ki67. The white lines indicate the presumptive boundary between the VZ and outside in the VZ (non-VZ). **(B)** Quantification of the number of Ki67+ cells in the VZ per 100 μm of cortical length. *N* = 3 to 6, *P = 0.0199, *df* = 2. **(C)** Representative immunostaining of E14.5 and E16.5 coronal brain sections for BrdU (red) and Ki67 (green). The bottom panel is a magnified image of the boxed area in the above picture. **(D)** Quantification of the percentage of BrdU+/Ki67− cells in the VZ negative for Ki67 per 100 μm of cortical length (right panel). *N* = 3 to 6, *P = 0.0015, *df* = 2. Error bars represent st. dev. Scale bar = 100 μm.

Reduced numbers of EOMES+ basal progenitor cells could result from reduced production by PAX6+ apical progenitor cells, increased cell death in this population or from a change in the balance between self-renewal and differentiation in this population. We could detect no difference in the number of cells undergoing apoptosis between wild-type and mutant embryos at E14.5, 16.5 or 18.5 as judged by staining with an antibody against the cleaved (activated) form of caspase-3 (Additional file 2: Figure S2). However, the VZ of the MBD3-null cortex showed reduced numbers of Ki67-positive cells at E16.5, but not at E14.5, emphasizing the presence of fewer total cycling progenitor cells in mutant brains between E15.5 and E16.5 (Figure 4A,B). This reduction in cycling cells, which is not associated with an increase in apoptosis, could be due to an increased frequency of cells exiting the cell cycle. To test this possibility, cortices from embryos that had been labelled with BrdU for 24 h were visualised for both BrdU incorporation and Ki67 reactivity. Those cells that had divided in the previous 24 h and subsequently exited the cell cycle would have incorporated BrdU, but would not stain for Ki67. Whereas a similar number of cells showed cell cycle exit at E14.5, by E16.5, the VZ of cKO embryos contained significantly more Ki67-negative cells that had taken up BrdU in the previous 24 h (Figure 4C,D).

The observed increase in cell cycle exit in the VZ could be the result of reduced numbers of EOMES+ basal progenitors (Figure 2) or an alteration in the cell cycle of PAX6+ apical progenitors. To examine the proliferation rate of the PAX6+ apical progenitor cell population at E16.5, we counted the percentage of PAX6 + BrdU+/BrdU total cells in the VZ using the samples described in Figure 3. These data show that fewer PAX6+ progenitors in Mbd3cKO embryos (4.1 ± 3.0 st. dev.) incorporated BrdU in the VZ between E15.5 and E16.5 when compared to WT embryos (17.9 ± 16.4 st. dev., *N* = 3 to 5, *P = 0.0200, *df* = 2) as measured by the percentage of BrdU + PAX6+/total BrdU cells in the VZ at E16.5 per 100 μm of cortical length. To measure the cell cycle distribution of the PAX6+ progenitors, we used flow cytometry on single PAX6+ cells isolated from E14.6 and E16.5 cortices (Additional file 3: Figure S3). We did not observe any change in cell cycle at E16.5 (Additional file 3: Figure S3A); however, at E14.5 Mbd3cKO, a higher percentage of PAX6+ progenitors were in S phase and fewer were in G1 (Additional file 3: Figure S3B). This is consistent with previously published reports showing that knockdown of the Mi-2β subunit of MBD3/NuRD results in a prolonged S-phase [26] and that Mi-2β regulates G1/S transition [27].

**Figure 5:**
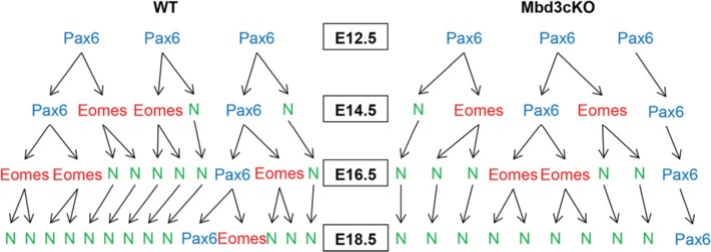
PAX6+ progenitors exiting the cell cycle result in decreased numbers of basal progenitors and neurons. This graphic illustrates how three representative PAX6+ apical progenitors in WT (left) and Mbd3cKO (right) mice make cell fate decisions over time. In the Mbd3cKO mice, a PAX6+ cell which exits the cell cycle without terminal differentiation into a neuron (far right) reduces the production of EOMES+ basal progenitors and neurons. This effect is small at first (E14.5) but increases in severity over time (E18.5). Note that in both WT and Mbd3cKO, the numbers of apical progenitors decreases over time, as expected, but at each time point, the numbers are comparable between the two groups.

Between E15.5 and 16.5, most divisions of PAX6+ apical progenitors are asymmetric: producing one neuron and one daughter progenitor cell, thereby maintaining the size of the PAX6+ apical progenitor population while still allowing for neuronal production [28]. Our data are consistent with a reduction in the rate of asymmetric divisions of PAX6-expressing apical progenitors, resulting in a decrease in neuronal production without a change in the size of the progenitor pool (Figures 2 and 5). Together, these data suggest an increase in the fraction of neural progenitor cells exiting the cell cycle in the absence of MBD3. Notably, this premature cell cycle exit does not appear to result in terminal neural differentiation of these progenitor cells, as they fail to migrate from the VZ (Figure 4C).

### Loss of MBD3 results in alterations in the proportions of different cortical neuronal subtypes

**Figure 6:**
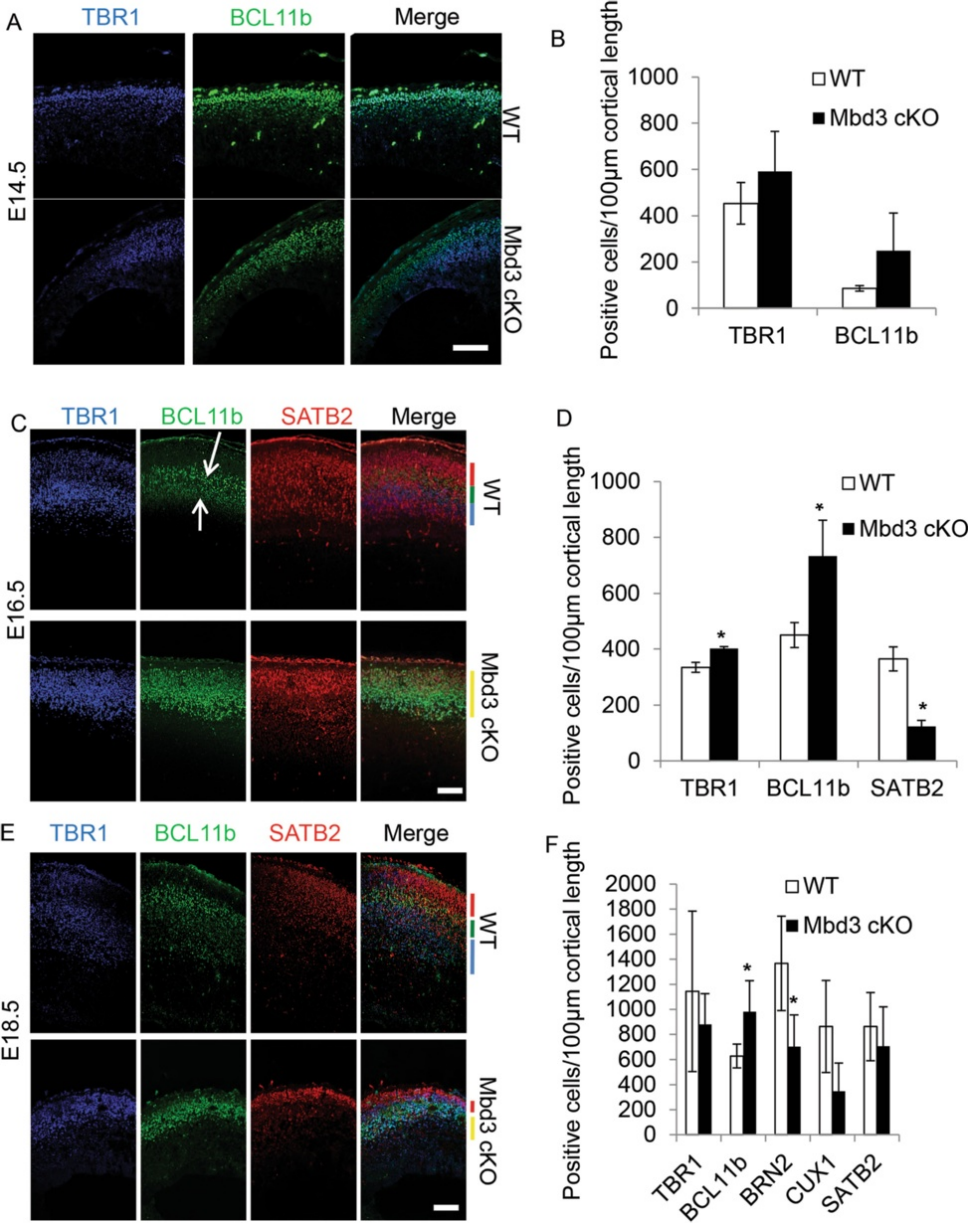
Altered neuron production and cortical layering in *Mbd3*-deficient brains. Representative immunostaining of coronal brain sections at E14.5 **(A)**, E16.5 **(C)** and E18.5 **(E)** for TBR1 (blue, layer 6), BCL11B (green, layer 5) and SATB2 (red, layers 2 to 4). Quantification of the number of stained cells per 100 μm cortical length at E14.5 **(B)**, E16.5 (**D**, *P = 0.0040, *df* = 2 [TBR1]; *P* = 0.0220, *df* = 2 [BCL11b]; *P* = 0.0010, *df* = 2 [SATB2]) and E18.5 (**F**, *P = 0.0100, *df* = 2 [BCL11b]; *P* = 0.0090, *df* = 2 [BRN2]). Scale bar = 100 μm; *N* = 3 to 6. Error bars represent st. dev. White arrows in **(B)** and **(C)** point to the BCL11B high and low expressing cells in WT brains, while in cKO brains, only the BCL11B high-expressing cells are present. Red, green and blue bars in **(C)** and **(E)**, WT merged images indicate distinct layers of cortical neurons present based on the staining. These layers do not appear or are reduced in the Mbd3 cKO brains (yellow bars, **(C)** and **(E)** merged images).

**Figure 7:**
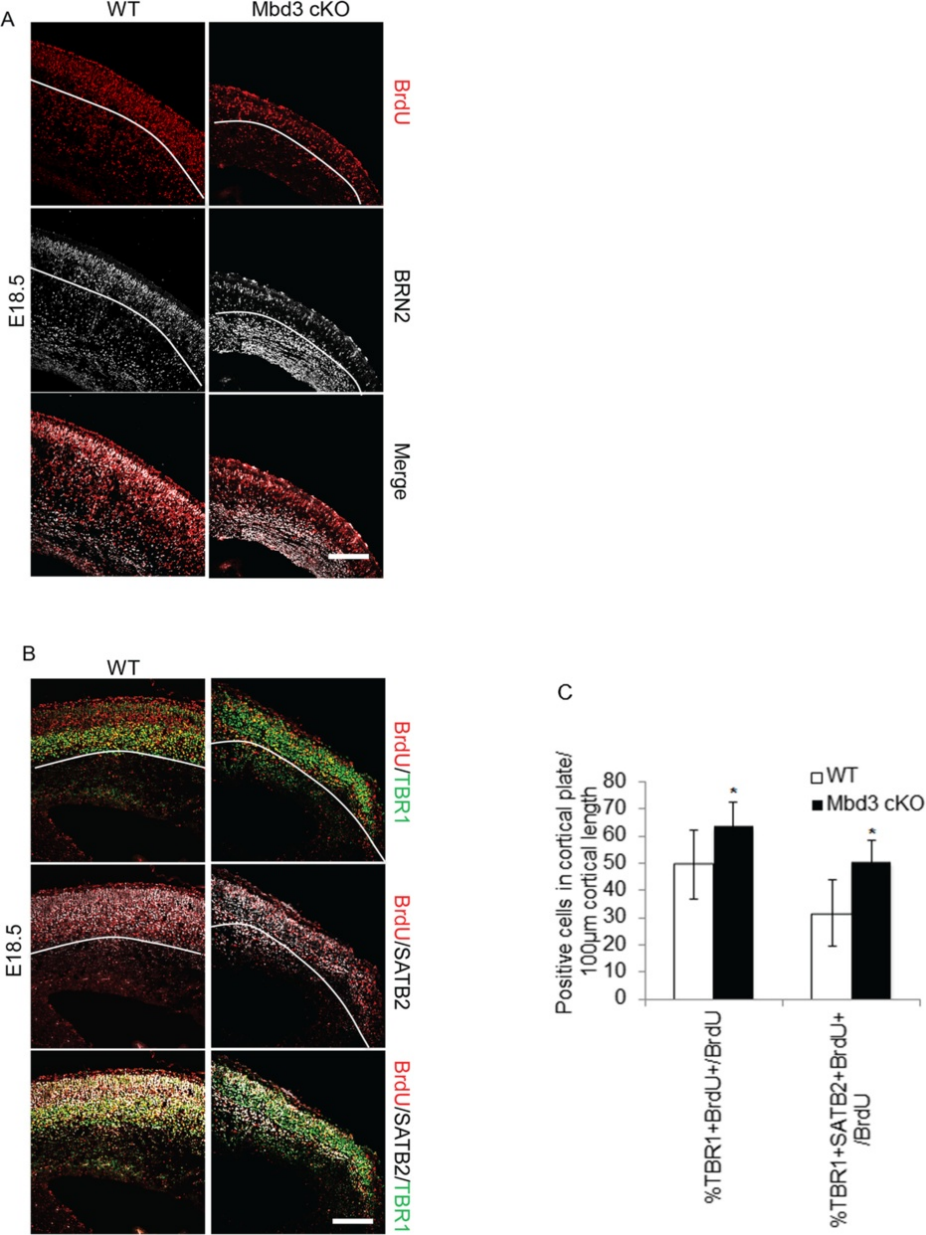
Failure of proper cortical neuron specification in the absence of MBD3. Pregnant mice were injected with BrdU once at E13.5 and the embryos examined at E18.5. **(A)** Representative immunostaining of E18.5 coronal brain sections for BrdU (red) and upper-layer neuronal marker BRN2 (white). **(B)** Representative immunostaining of E18.5 coronal brain sections for BrdU (red), TBR1 (green) and SATB2 (white). **(C)** Quantification of the percentage of cortical plate BrdU+ cells which are also positive for TBR1 (*P = 0.0210, *df* = 2) or positive for both TBR1 and SATB2 (*P = 0.0016, *df* = 2) per 100 μm of cortical length. The white lines indicate the presumptive lower boundary of the cortical plate. Scale bar = 100 μm; *N* = 3 to 6. Error bars represent st. dev.

To quantify the timing of appearance and the relative numbers of different cortical neuronal types, the expression of transcription factors specifically expressed in different neuronal populations was analysed in wild-type and mutant embryos at three stages of cortical development (E14.5, E16.5 and E18.5). Deep-layer neuron transcription factors included TBR1 (layer 6) and BCL11B/CTIP2 (layer 5), and upper-layer neurons were identified by expression of SATB2, CUX1 and BRN2 (Figure 6 and 7A, Additional file 4: Figure S4). MBD3 cKO cortices contained normal proportions of TBR1 and BCL11b-expressing deep-layer neurons at E14.5 (Figure 6A,B). However, at E16.5, the MBD3 cKO cortex contained significantly more TBR1 and BCL11B-expressing cells and significantly fewer SATB2-expressing cells than WT controls (Figure 6C,D). In contrast, we saw no defects in the earliest born neuronal population (layer 1), as judged by Reelin staining, at E16.5 (Additional file 5: Figure S5). By E18.5, the MBD3 cKO cortex continued to have more BCL11b expressing cells but only showed a reduction in the number of upper-layer cells expressing BRN2 (Figures 6E,F and 7A). These data support previous studies [19,20] suggesting that MBD3/NuRD may be involved in suppressing BCL11B in SATB2 expressing neurons. To confirm this finding, we counted the %SATB2 and BCL11B double-positive cells/total SATB2-positive cells. As expected, Mbd3 cKO mice at E16.5 had a significantly higher %SATB2+ BCL11B+/SATB2+ cells per 100 μm cortex compared to WT mice (WT: 35.1 ± 14.6 st. dev., cKO: 63.5 ± 24.8 st. dev., *P* = 0.0006, *N* = 4, *df* = 3).

In addition to a reduced number of cortical neurons, MBD3-mutant brains also exhibited a defect in cortical lamination. While wild-type E16.5 and 18.5 brains contained the distinct populations of BCL11B-high (layer 5) and BCL11B-low (layer 6) expressing cells associated with proper cortical architecture [29], cKO cortices contained only a BCL11B-high expressing population (white arrows in Figure 6C,E). Further, while normally laminated wild-type cortices had distinct layers of cells neurons expressing singly either BCL11B (layer 5), SATB2 (layer 2/3) or TBR1 (layer 6) (Figure 6) [3,29], no such organisation was evident in mutant cortices. Instead, cells expressing the different markers were intermingled (coloured lines in Figure 6C,E). Although MBD3 is dispensable for TBR1 and BCL11b-expressing early neuronal production, proper differentiation of later, SATB2 and BRN2-expressing upper-layer neurons and appropriate cortical lamination require the function of MBD3/NuRD.

### Deep-layer neuron differentiation is extended in the MBD3-null cortex

Given the alterations in the relative numbers of BCL11b and SATB2/BRN2 expressing neurons in the MBD3-null cortex during cortical development, the timing of the production of upper-layer neurons was assessed by BrdU birthdating. Around E13.5, neurogenesis switches from deep-layer neuron production to upper-layer neuron production [30]. Therefore, neuronal birthdates were studied in embryos labelled at E13.5 with a single dose of BrdU and analysed at E18.5 for expression of transcription factors specific to upper-layer (BRN2 and SATB2) and deep-layer (TBR1) neurons (Figure 7). Consistent with the decrease in total numbers of BRN2-expressing upper-layer neurons in the MBD3-null cortex E18.5 (Figure 6F), a significantly smaller proportion of neurons produced at E13.5 express the upper-layer neuronal marker BRN2 in MBD3-deficient brains (Figure 7A, %BRN2 + BrdU+/BrdU+ in cortical plate per 100 μm of cortical length: WT: 50.6 ± 16.4 st. dev., cKO: 29.9 ± 10.5 st. dev., *P = 0.0016, *df* = 2). Further, an increased proportion of these younger neurons express both the deep-layer marker TBR1 and upper-layer marker SATB2 (Figure 7B,C). These data indicate that more neurons born at E13.5 in the MBD3-null cortex do not have a well-defined cortical identity compared with their WT counterparts. The co-expression of TBR1 and SATB2 is consistent with aberrant terminal differentiation of later born neurons, rather than a simple extension of the period of deep-layer genesis by progenitor cells.

### Regulation of neurogenic gene expression programmes by the MBD3/NuRD complex

MBD3 is a structural component of NuRD, a transcriptional co-repressor complex. We therefore expected that the phenotypes observed in cKO mice would arise from failure to properly regulate gene expression. In order to generate a profile of MBD3/NuRD-dependent gene expression in neural progenitors during development, whole-genome expression analysis was carried out on an enriched population of progenitor cells laser capture microdissected from the VZ of WT and mutant cortices at E12.5, E14.5 and E16.5. Embryonic day 12.5 is prior to the emergence of overt phenotypes and was expected to yield primary gene misexpression events in cortical progenitor cells. By E16.5, the mutant phenotypes were fully apparent and thus would be expected to display both primary and secondary gene misexpression events. Consistent with our previous work in ES cells, primary gene expression changes in the absence of MBD3 are subtle, that is, less than fourfold (ArrayExpress accession number E-MTAB-2778) [17,31].

**Figure 8:**
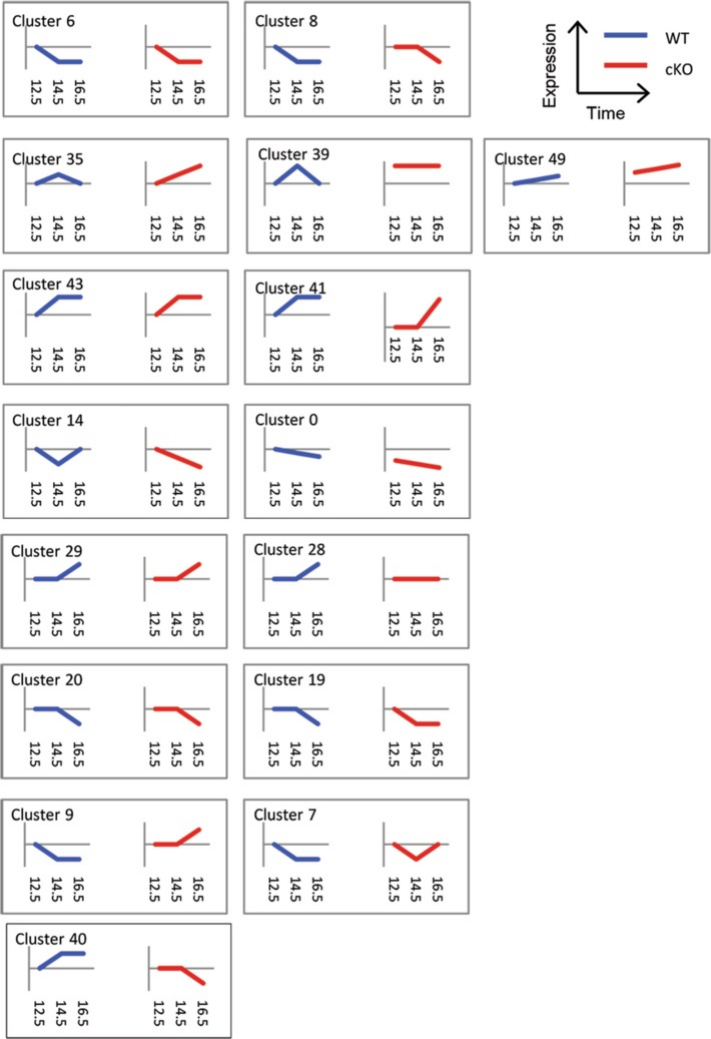
Total output from the STEM programme. Significant clusters identified by STEM analysis. For each panel, the expression in wild-type embryos is shown in blue on the left and in mutant embryos in red on the right. Developmental time (E12.5, E14.5 and E16.5) is plotted on the *x*-axis and expression levels on the *y*-axis in arbitrary units. For each cluster, the data are plotted along the *x*-axis as WT12.5, WT14.5 and WT16.5 (blue) and cKO12.5, cKO14.5 and cKO16.5 (red).

To get a picture of how gene expression patterns change over the neurogenic period of brain development, we compared gene expression data across all three developmental time points in wild-type and mutant embryos using the short time-series expression miner (STEM) programme [32]. This analysis resulted in the identification of 16 different gene expression profiles that were significantly overrepresented in our expression data (see Figure 8). Four of these 16 profiles show the mutant samples behaving similarly to wild-type samples (Clusters 6, 29, 43 and 20). The remaining 12 profiles all show some degree of difference between wild-type and mutant brains. These profiles indicate that, like in ES cells, MBD3/NuRD does not simply function as an on/off regulator of gene expression during neural development but is important for maintaining levels of expression to within a particular range as transcriptional programmes are activated/deactivated during neural development [17,33].

**Figure 9:**
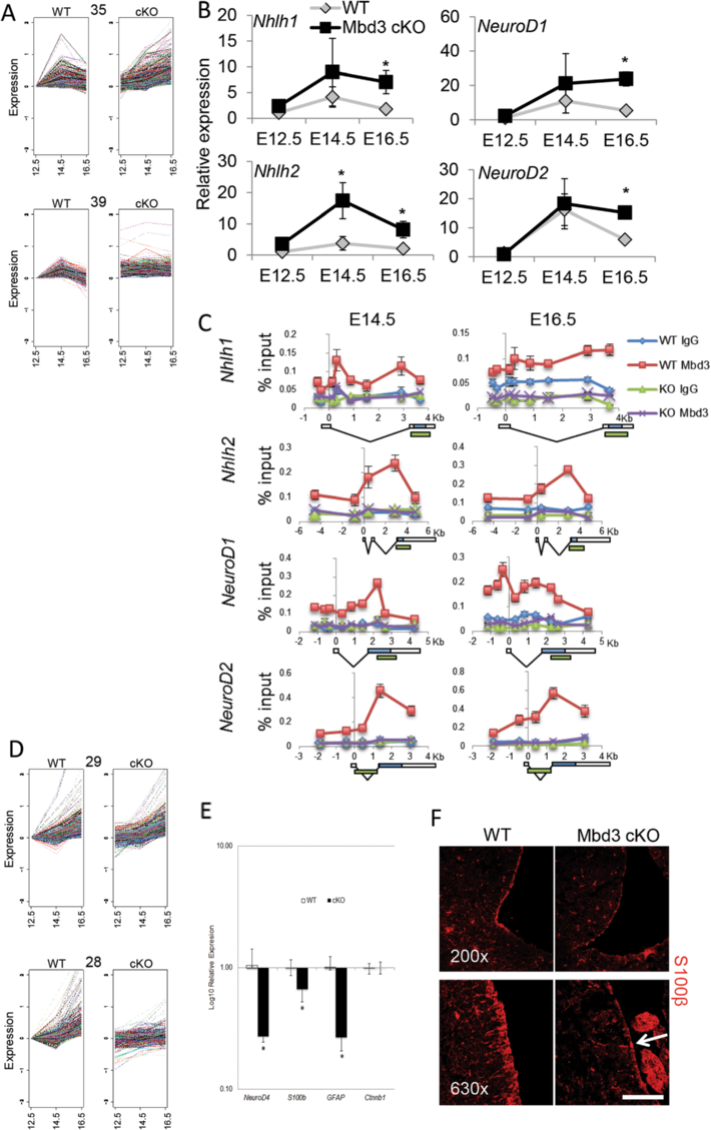
MBD3/NuRD modulates gene expression patterns during mammalian neurogenesis. **(A)** Expression of genes found in clusters 35 and 39 are plotted individually for wild-type (left) and Mbd3 cKO (right) samples. **(B)** Relative expression of indicated genes in WT and mutant progenitors at E12.5, E14.5 and E16.5. In all cases, expression values are plotted relative to WT expression at E12.5. *N* = 3 to 4. For wild-type *vs*. mutant samples at E14.5 for *Nhlh2* *P = 0.0066, *df* = 2. For wild-type *vs*. mutant samples at E16.5 *P = 0.0032, *df* = 2 (*Nhlh1*); *P* = 0.0002, *df* = 2 (*NeuroD1*); *P* = 0.0403, *df* = 2 (*Nhlh2*); *P* = 0.0001, *df* = 2 (*NeuroD2*). **(C)** Chromatin immunoprecipitation (ChIP) was performed using an anti-Mbd3 antibody or rabbit IgG control in wild-type (WT) and conditional knockout (KO) cortices at E14.5 and E16.5. Immunoprecipitates were probed with primer pairs (listed in Table 3) located across the indicated genes and plotted as percentage of input (*y*-axis; error bars represent st. dev.). Numbers across the *x*-axis indicate distance relative to the transcription start site for indicated genes. Gene structure is shown below the graphs: white boxes represent non-coding regions, blue boxes represent coding regions, and green boxes indicate the presence of putative enhancer regions based upon histone ChIP data available on mm9 from the UCSC genome browser. **(D)** Expression of genes found in clusters 28 and 29 are plotted individually for wild-type (left) and cKO (right) samples. **(E)** Relative expression of indicated genes in wild-type and mutant brain samples from E16.5. *N* = 3; *P = 0.0004 (*NeuroD4*), *P* = 0.0026 (*S100β*), *P* = 0.0001 (*Gfap*), *df* = 2; error bars represent st. dev. **(F)** Representative immunostaining of E18.5 coronal brain sections for S100β (red). White arrow indicates a rare, positively stained cell in the Mbd3 cKO sample Scale bar = 100 μm at 200× magnification and 50 μm at 630× magnification.

Between E12.5 and E16.5, the principal output of the neurogenic programme is neuronal, and concordantly, progenitor cells will show some expression of genes involved in neuronal specification. By E16.5, this programme begins to switch from predominantly neural production towards glial cell production [34], which is associated with a decrease in expression of neurogenic genes. Clusters 35 and 39 both depict gene expression patterns in wild-type samples consistent with this scenario, where genes show increased expression from E12.5 to E14.5, but then decreased expression by E16.5 (Figures 8 and 9A, gene lists in Additional file 6: Table S1 and Additional file 7: Table S3). However, the expression patterns of genes in these clusters differ in mutant cells. Cluster 39 genes are expressed at an elevated level at all stages, possibly indicating a lack of transcriptional control of these genes. Cluster 35 genes show normal increase in expression from E12.5 to E14.5, but instead of then decreasing again by E16.5, the expression of these genes remains high at E16.5. This lack of proper expression only at E16.5 indicates a specific failure to maintain the appropriate levels of gene expression in response to developmental cues, rather than a general lack of regulation, as with Cluster 35 genes. Cluster 35 shows a highly significant enrichment for genes associated with gene ontology (GO) terms involving neurogenesis (Additional file 8: Table S2, *P* = 1.91 × 10^−4^) consistent with the peak and decline of neurogenesis from E12.5 to 16.5. Cluster 39 shows a less significant enrichment of genes associated with neurogenesis (*P* > 0.01) but rather shows enrichment for genes involved in cytoskeleton organisation (*P* = 6.99 × 10^−4^) and chromatin organisation (*P* = 2.7 × 10^−3^) (Additional file 9: Table S4). This pattern of gene expression identified in Cluster 35, characterised by a failure to appropriately decrease neurogeneic gene expression at the onset of gliogenesis in *Mbd3*-mutant brains, implicates MBD3/NuRD function in the execution of the neural-to-glial developmental switch.

The expression of some neurogenic bHLH transcription factors has been shown to peak at the height of neurogenesis (E14.5) and their downregulation is necessary for gliogenesis to begin [29,35,36]. One such transcription factor, *Nhlh1*, was included in Cluster 35 and was also in the list of genes upregulated in E16.5 mutant brains (ArrayExpress accession number E-MTAB-2778). To verify this gene expression pattern, we determined the expression patterns of four neurogenic bHLH transcription factors, *Nhlh1*, *Nhlh2*, *NeuroD1* and *NeuroD2*, over the course of neurogenesis in progenitors from WT and cKO embryos by quantitative reverse transcriptase polymerase chain reaction (qRT-PCR) (Figure 9B). In both WT and Mbd3 cKO brain samples, all four genes show a peak of expression at E14.5 and a subsequent reduction in expression by E16.5. This indicates that, in the absence of MBD3/NuRD activity, neural progenitors are capable of responding to the developmental signals promoting expression of these genes at E12.5 and are also able to respond to signals that result in a reduction of gene expression by E16.5. The expression levels of *Nhlh2* are elevated at E14.5 in mutant samples compared to those seen in wild-type samples, suggesting that disruptions in neurogenesis may begin even before we are able to detect the phenotype. Additionally, despite three of the four genes showing a reduction of expression after E14.5, all four genes remain expressed at elevated levels at E16.5 in the absence of MBD3/NuRD activity. Chromatin immunoprecipitation (ChIP) analyses of dissected cortices at E14.5 and E16.5 showed that Mbd3 is indeed associated with predicted regulatory regions in all four of these loci in wild-type embryos at both time points, consistent with direct regulation of these neurogeneic genes by the MBD3/NuRD complex (Figure 9C). Thus, despite apparently being able to respond to inductive signals, a lack of MBD3/NuRD activity results in overexpression of neurogenic factors at later stages of neural development.

Downregulation of the neurogenic gene expression programme is normally associated with activation of genes important for initiation of gliogenesis during normal brain development, with the peak of gliogenesis occurring in early postnatal stages [34]. Importantly, histone deacetylase activity has been shown to be important for this neurogenic to gliogenic switch [37]. Clusters 28 and 29 identify two sets of genes that show little change between E12.5 and E14.5, but activation by E16.5 (Figure 9D, gene lists in Additional file 10: Table S5 and Additional file 11: Table S7). However, for the Cluster 28 genes, there is a failure to increase expression levels at E16.5 in the mutant brains. Included in this cluster are genes implicated in glial cell development, including *Gfap* [38] and *Neurod4* [39] (Additional file 12: Table S6, GO analysis found no significant enrichment of terms in cluster 29). Reduced expression of these genes in E16.5 mutant brains was subsequently verified by qRT-PCR (Figure 9E). E18.5 mutant brains displayed a considerable reduction in cells expressing the glial cell marker S100β compared to wild-type brains (Figure 9F), consistent with a defect in gliogenesis in *Mbd3*-deficient mice. As these mice died at birth, prior to the peak of glial cell production, it was not possible to determine whether this defect in gliogenesis seen at E18.5 was due to a failure or a delay in gliogenesis. Nevertheless, these data support the conclusion that MBD3/NuRD activity functions to ensure the successful execution of developmental transitions during mammalian neurogenesis.

## Discussion

Neural development proceeds in a regimented fashion during mammalian development, with different cell types being produced in a strict temporal order [1]. Each cell type is specified by a distinct transcriptional programme such that maintaining correct expression levels of specific transcriptional programmes is essential for proper execution of each distinct developmental transition. Here, we use genetic analyses to show that transcriptional modulation by the MBD3/NuRD complex is essential for the proper execution of a number of developmental transitions during mammalian neurogenesis.

In ES cells, the NuRD component protein MBD3 is dispensable for self-renewal but plays an important role during lineage commitment [12]. In contrast, during neurogenesis, MBD3 is required neither for proliferation nor differentiation of neural progenitor cells during embryonic development, but rather ensures proper execution of lineage-determination programmes. This is consistent with published reports of MBD3/NuRD function in other somatic stem cell types [14,16]. Our observations also indicate that fewer PAX6+ apical progenitors are dividing at later stages in *Mbd3* cKO brains. We cannot exclude the possibility that the same number of PAX6+ apical progenitors are dividing but with an increase in cell cycle length, although the decreased neurogenesis and cell cycle profiles at E16.5 argue against this. Interestingly, despite equal numbers of PAX6+ progenitors going through cell cycle (at any stage, Figures 3A,B and 4), the cell cycle distribution is abnormal at E14.5. Based on this observation, we speculate that there is a subtle defect in cell cycle dynamics at E14.5 which leads to more cells dropping out of cell cycle by E16.5.

During normal neurogenesis, apical progenitor cells lengthen their cell cycle and switch from primarily symmetric cell divisions to primarily asymmetric divisions. The asymmetric divisions produce both basal progenitors and neurons. Our observations of fewer BrdU+/PAX6+ apical progenitors and more BrdU+/Ki67− cells indicate that the PAX6+ apical progenitors are unable to respond to the signals regulating symmetric *vs*. asymmetric divisions. They instead drop out of cell cycle and fail to produce sufficient EOMES+ basal progenitors and neurons by asymmetric divisions. This is supported by our observation that mutant cortices show evidence of increased cell cycle exit after E15.5 without a subsequent increase in the number of differentiated neurons (Figures 3B and 4). Rather, lack of MBD3 resulted in decreased numbers of both EOMES+ basal progenitors and neurons by E16.5. The result of this is illustrated in Figure 5. In WT mice, PAX6+ progenitors increase their asymmetric divisions over time, producing EOMES+ basal progenitors and neurons leading to an increase in the number of neurons while decreasing the size of the progenitor pool (left panel). Early cell cycle defects in *Mbd3* cKO PAX6+ progenitors leads to more and more cells exiting cell cycle without differentiating. While the number of PAX6+ progenitors still decreases over time, as in WT, there is no corresponding increase in EOMES+ basal progenitors and neurons (right panel).

MBD3 is expressed in a subpopulation of cortical plate neurons, the position of which indicates that it is primarily expressed in upper-layer neurons (Figure 1A). Without MBD3, proper specification of SATB2- and BRN2-expressing upper-layer neurons is compromised (Figure 6). These observations are in agreement with previous studies reporting that MBD3/NuRD subunits interact with the transcription factor SATB2 in order to repress expression of *Bcl11b* and thereby allow the transition between deep-layer and upper-layer neuron production [19,20]. Our data support this conclusion and suggest that, in addition to producing fewer neurons, the superficial neurons that are produced co-express deep-layer and upper-layer neuronal markers (Figure 7). That accounts for the increased numbers of deep-layer marker expressing cells we observed (Figure 6). Together, these data support previously reported roles for the MBD3/NuRD complex in maintaining distinct deep and upper neuronal layers [19,20].

Analysis of global gene expression patterns in neural progenitors from E12.5 to E16.5 provided further evidence for MBD3/NuRD’s involvement in regulating developmental transitions in the brain. It is notable that the genes we see expressed in our study are not those which are normally detectable at the protein level in neural progenitors. Although we were able to enrich for neural progenitors using the laser capture microdissection technique, we cannot exclude the possibility that some immature or mature neurons were also captured during this procedure. Nevertheless, unbiased clustering of gene expression patterns revealed 12 different scenarios in which gene expression changes over three developmental time points occur aberrantly in *Mbd3*-deficient embryos. This indicates that neural progenitors can respond to the developmental cues regulating both activation and repression of transcription in the absence of a functional MBD3/NuRD complex. However, despite showing some reduction in expression levels between E14.5 and E16.5, in the absence of MBD3/NuRD activity, the expression levels of some proneural genes are not reduced to wild-type levels at E16.5. Our observation that MBD3/NuRD directly regulates these genes (Figure 9B and C) is consistent with a recent study showing that CHD4 (an MBD3/NuRD complex component) directly regulates *Nhlh1* in the adult mouse cerebellum [40].

Unbiased clustering also identified a set of genes that fails to be appropriately activated at E16.5, including a number of genes indicative of a gliogenic transcriptional programme. *Mbd3* cKO mice die at birth, so we were not able to completely assess gliogenesis in this system. However, decreased staining for the early gliogenic marker S100β at E18.5 in mutant brains and reduced expression of genes associated with gliogenesis are consistent with a role for MBD3/NuRD in regulating the neurogenic to gliogenic switch. Therefore, while we see no evidence that the persistent expression of the proneural genes prolongs neurogenesis, it may be impeding the switch to gliogenesis.

In ES cells, the MBD3/NuRD complex controls the dynamic range of gene expression, the function of which is to maintain cells in a state that is competent to respond to the presence or absence of specific differentiation cues [17,41,42]. Our gene expression analyses are consistent with MBD3/NuRD functioning similarly in neural cells. When comparing gene expression in wild-type and cKO brains, we find significant misexpression of relatively few genes and the level of gene expression changes are very modest (<fivefold). In contrast, loss of the PRC2 component protein EZH2 in cortex resulted in misexpression of more than 1,000 genes [25].

## Conclusions

We propose that MBD3/NuRD acts in the developing brain to keep the levels of pro-neurogenic gene transcription in check so that cells can properly respond to developmental cues and, when appropriate, switch to a different transcriptional programme. In the absence of MBD3/NuRD we find that cells are able to respond to developmental signals, but are unable to appropriately down-regulate expression of genes that would be inappropriate in the new lineage to be specified (for example, Figures 7C,D and 9). Consequently, progenitor cells are unable to properly execute their normal lineage decisions, resulting in alterations in progenitor cell homeostasis and an inability to generate the normal spectrum of neuronal subtypes. Notably, this activity for MBD3/NuRD in down-regulating gene expression during developmental transitions has previously been described in early mouse development, in ES cells, in nematodes and in plants [8,17,43,44].

## Methods

### Mice

All mouse husbandry and experimentation was approved by the Animal Welfare and Ethical Review Body, University of Cambridge, and performed under a licence granted by the UK Home Office. The generation of mice expressing an *Mbd3* exon 1 floxed allele (hereafter referred to as flox) has been described [45]. Nestin-Cre (N-Cre) expressing mice were a gift from Francois Tronche and have been previously described [22]. N-Cre mice were mated with *Mbd3*^*flox*/*+*^ mice to generate *N-Cre:Mbd3*^*flox/+*^ mice. The genotypes of all offspring were recorded at several embryonic stages (Table 1). The *N-Cre:Mbd3*^*flox/flox*^ genotype is embryonic lethal in the majority of cases; any mice with this genotype born alive died within 24 h of birth (Table 1). No difference was observed in the expected ratios for the other genotypes, including *N-Cre:Mbd3*^*flox/+*^. For the purposes of this paper, therefore, we will refer to all mice with the *N-Cre:Mbd3*^*flox/flox*^ genotype as conditional knockouts (cKO) or mutant and all other genotypes as WT. Both male and female mice were used in this study. For neuronal output and birth dating experiments, pregnant mice were injected with a single dose of BrdU (40 mg/kg, Sigma, Gillingham, UK) at E13.5 or 15.5.

**Table 1:** ***N-Cre: Mbd3*** ^***flox/flox***^ **peri-natal mortality**

	**Percentage (%)**				
**d.p.c.**	**N-Cre: Mbd3** ^**Flox/+**^	**Mbd3** ^**Flox/+**^	**Mbd3** ^**Flox/Flox**^	**N-Cre: Mbd3** ^**Flox/Flox**^	***N***
12.5	25	12.5	25	20.8	24
14.5	25.9	25.9	14.8	22.2	27
15.5	50	25	12.5	12.5	16
16.5	17.5	35	29.1	14.6	57
17.5	36.4	18.2	22.7	22.7	22
Postnatal	28.4	40.8	29.6	0.8^a^	250

^a^All *N-Cre:Mbd3* ^*Flox/Flox*^ mice born live were found dead within 24 h after birth. Genotypes of pups and embryos produced from N-Cre(Flox/+) × (Flox/Flox) intercrosses recovered at indicated time points.

### Immunohistochemistry

Whole heads (E12.5 and E14.5 embryos) or dissected whole brains (E16.5 and E18.5 embryos) were fixed in 4% paraformaldehyde (Sigma, Gillingham, UK) for 24 h (for E12.5 and E14.5 embryos) or 48 h (for E16.5 and E18.5 embryos) and then switched to a 30% sucrose/PBS solution. Coronal sections were cut at a thickness of 10 μm and sagittal sections at a thickness of 4 μm. Sections from wild-type and mutant embryos were mounted on the same slide so that the wild-type section could act as a positive control for the mutant section during antibody staining.

MBD3 staining was performed using a Ventana Discovery® (Ventana Medical Systems Inc., Tuscon, AZ, USA) according to the manufacturer’s instructions. Both the primary (goat anti-MBD3, 1:100, sc-9402, Santa Cruz Biotechnologies, Santa Cruz, CA, USA) and the secondary (biotinylated anti-goat, 1:500, Vector Laboratories, Peterborough, UK) antibodies were titrated manually. Haematoxylin was used as a counterstain.

Immunofluorescent staining was performed on coronal frozen sections with antigen retrieval by boiling three times for <1 min in 0.01 M citrate buffer (pH 6.0) containing 0.05% Tween-20. For slides stained using anti-BrdU, a 1-h incubation in 2 N HCl at 37°C was also performed. Primary antibodies used are as follows: rat anti-BrdU, 1:100, ab6326, Abcam (Cambridge, UK); rabbit anti-BRN2, 1:100, sc-6029, Santa Cruz Biotechnology (Santa Cruz, CA, USA); rabbit anti-Caspase-3 (cleaved), 1:100, 9664S, Cell Signalling Technologies (Hitchin, UK); rat anti-BCL11B, 1:500, ab18465, Abcam (Cambridge, UK); rabbit anti-CDP (CUX1), 1:200, sc-13024, Santa Cruz Biotechnology (Santa Cruz, CA, USA); rat anti-phosphorylated Histone H3, 1:500, ab10543, Abcam (Cambridge, UK); rabbit anti-Ki67, 1:100, Vector Laboratories (Peterborough, UK); rabbit anti-PAX6, 1:500, ab2237; Millipore (Watford, UK); mouse anti-Reelin, 1:100, mab5384, Millipore (Watford, UK); mouse anti-SATB2, 1:100, ab51502, Abcam (Cambridge, UK); rabbit anti-S100β, 1:100, Z0311, Dako Cytomation (Ely, UK); rabbit anti-TBR1, 1:250, ab31940, Abcam (Cambridge, UK); rabbit anti-EOMES, 1:500, ab23345, Abcam (Cambridge, UK); mouse anti-TuJ1, 1:50, MMS-435P, Covance (Maidenhead, UK). Secondary antibodies used are as follows: Cy3 conjugated donkey anti-rat, 712-165-153, Jackson Immunoresearch Laboratories Inc. (Newmarket, UK); Alexa-Fluor^©^ - 488 (A21206) or 555 (A31572) conjugated donkey anti-rabbit and Alexa-Fluor^©^ - 488 (A21202), 555 (A31570) or 647 (A31571) conjugated donkey anti-mouse, Life Technologies (Paisley, UK).

### Image acquisition and analysis

MBD3-stained sections were visualised using a Zeiss axioimager bright field microscope (Welwyn Garden City, UK). Sections with immunofluorescent staining were visualised using a Leica Microsystems SP5 TCS confocal microscope (Leica Microsystems, Milton Keynes, UK). Quantification of the number of positively stained cells for each antibody was carried out on confocal microscope images with ImageJ software and the ITCN plug-in [25]. One or two matched WT and cKO littermates from two to three different litters were chosen for analysis. Three non-consecutive slides from each sample were chosen for staining. A schematic showing where in the brain each picture was taken is shown in Additional file 1: Figure S1A. The three sections chosen per mouse were rostral, middle and caudal sections; the results are presented as each area separately (Figure 1), averaged mid-cortical sections only (Figures 2B,D and 6) or averaged over all three areas (Figures 2F,G, 4, and 7). The images shown are either anterior (Figure 2E), middle (Figures 1, 2A,C, 3, 6, and 9) or caudal (Figures 4 and 7). Briefly, an intensity threshold for positive staining was chosen for each antibody. A region of interest that was 100 μm along the cortical length was designated (either the whole cortex, cortical plate, VZ or non-VZ, as indicated), and all cells with staining above the threshold were counted. For the BrdU/PAX6 staining, a very high threshold for BrdU positive staining was chosen so as to exclude weakly positive basal progenitors from the counts (indicated in the figure as BrdU++). Measurements are reported as mean ± the standard deviation. GraphPad QuickCalcs online tool (http://www.graphpad.com/quickcalcs/index.cfm) was used to for all statistical analysis. Grubb’s test was used to detect and remove statistical outliers. An unpaired two-tailed student’s *t*-test was used to determine the statistical differences between WT and Mbd3 cKO.

### Cell cycle analysis

Cortices were dissected from E14.5 and E16.5 embryos and a single-cell suspension made before fixing in 70% ethanol for 30 min. The fixed cells were washed twice in PBS +10% foetal calf serum and re-suspended in 1 ml PBS+ 10% serum. An equal volume of re-suspended cells was taken from each sample and pooled. This pool was the split into three parts: a primary antibody only control, a secondary antibody only control and a propidium iodide only control. The samples were stained with a 1:500 dilution of anti-PAX6 antibody and an AlexaFluor 647 conjugated anti-rabbit secondary following the Abcam direct flow cytometry protocol (http://www.abcam.com/index.html?pageconfig=resource&rid=11380; Cambridge, UK). Before analysis, 200 μl propidium iodide (50 μg/ml, Sigma, Gillingham, UK) was added to each sample and controls. Flow cytometry was performed using a CyAn ADP Analyzer (Beckman Coulter Inc., High Wycombe, UK). A gate was set to PAX6-positive cells only. The data were then exported into FlowJo software (Tree Star Inc., Ashland, OR, USA), and the cell cycle was analysed using the software’s pre-set Dean-Jett-Fox method. An unpaired two-tailed *t*-test using the GraphPad QuickCalcs online tool was performed to detect significant differences between genotypes.

### LCM and RNA extraction

Coronal brain sections of 4 WT and 4 cKO E12.5, E14.5 and E16.5 embryos were used for laser capture microdissection using a Zeiss (Welwyn Garden City, UK) PALM Microbeam laser capture system as per the manufacturer’s instructions. The VZ from the left and right hemispheres from 3–4 sections per slide were dissected into adhesive cap tubes and incubated overnight at 55°C in 75 μl Proteinase K buffer (100 mM Tris pH7.5, 200 mM NaCl, 2 mM EDTA, 1% SDS), 65 μl water and 250 μg Proteinase K. The samples were treated with 3.75 μl of DNAseI and 15 μl buffer RDD from the RNAse-free DNAse Kit (Qiagen) for 10 minutes at room temperature. RNA was extracted using the Qiagen RNeasy Plus micro kit, as per the manufacturer’s instructions.

### Microarray and data analysis

RNA amplification (Ovation pico WTA system, Nugen, Bemmel, The Netherlands) and hybridization to MouseWG-6 *vs*. 2 Illumina arrays was performed by Cambridge Genomic Services (University of Cambridge, Department of Pharmacology). Expression data were preprocessed using the R package ‘lumi’ [46]. A variance stabilising transformation, robust spline normalisation and QC were performed on the expression data. To test for differential expression, the statistical method limma was used [47]. Multiple testing correction based on the false discovery rate (FDR) was performed. F-statistics from the Bayesian adjusted linear models were used to determine significant differential expression. The FDR method used was ‘nestedF’ and the minimum log fold change required was +/− 0.5 (ArrayExpress accession number E-MTAB-2778). The software programme STEM was used to cluster and analyse the gene expression data [32]. The STEM programme calculates the significance of a cluster based on the ratio of the number of assigned genes versus the number of expected genes to a profile. The *P* value threshold used was 0.05, which was adjusted for multiple testing using the Bonferroni correction. GO analyses were performed using DAVID (http://david.abcc.ncifcrf.gov/summary.jsp). DAVID uses a modified Fisher Exact p-Value and the threshold used was 0.05.

### qRTPCR

RNA from LCM was used to make cDNA using the transcriptor first-strand cDNA synthesis kit (Roche, West Sussex, UK) following the manufacturer’s instructions. The resulting cDNA was used in triplicate SYBR green reactions (Life Technologies, Paisley, UK). Primers are listed in Table 2. *Ppia* expression was used as a control. Relative expression values are reported as mean ± the standard deviation. For comparisons between cKO and WT, an unpaired two-sided *t*-test was performed.

**Table 2:** **Primers used for gene expression analyses**

**Sequence (5′ to 3′)**	**Gene**
TTGACTGCCGAGAAGTTGTG	Ctnnb1
CTGGCATTTTGGAGAGGAAG	Ctnnb1
TTTCTCCAACCTCCAGATCC	Gfap
CCCGCATCTCCACAGTCTT	Gfap
AAAGACCTGCAGCACTTGAG	Nhlh1
TCCAGCACATGGTTCAGGTA	Nhlh1
GTGTCGGACCTAGAGCCAGT	Nhlh2
CAGGTTGAAAGCCTCCACTC	Nhlh2
TGGGTCTTGGAGTAGCAAGG	NeuroD1
GGAGGAGGAGGATCAAAAGC	NeuroD1
CGAAGAAACGCAAGATGACC	NeuroD2
TTCTGCGTCTTGGAGTAGCA	NeuroD2
ACCCCGGGAAAGAGAATCTA	NeuroD4
TCCACCATGTCCTTGGATTT	NeuroD4
GGACACTGAAGCCAGAGAGG	S100β
TTCAGCTTGTGCTTGTCACC	S100β
CACGGGGGCCTGTCTCCAGA	Ppia
GTCAGGCACGTCTGTGGGCC	Ppia

### Chromatin immunoprecipitation

Cortices were dissected from E14.5 or E16.5 brains. Two (E16.5) to three (E14.5) cortices of like genotype were dissociated together in 1 ml of PBS using a Dounce homogeniser. Formaldehyde was then added to a final concentration of 1% and allowed to fix for 10 min. Glycine was then added to a final concentration of 0.125 M. ChIP was then performed using standard methods, as described (Reynolds *et al*. [17]), using an anti-Mbd3 antibody (rabbit anti-Mbd3, A302-528A, Bethyl Laboratories, Montgomery, TX, USA) or a rabbit IgG control (Invitrogen, Carlsbad, CA, USA). ChIPs were performed a minimum of three times, and qPCR was carried out in triplicate using primers indicated in Table 3.

**Table 3:** **Primers used for ChIP**

**Primer name**	**Sequence (5′ to 3′)**
Nhlh1 -455 F	AGGGAGGACCTTCAGGTTAC
Nhlh1 -455R	TTTCATTCTGAGTCCCAGCG
Nhlh1 -298 F	TGCTACCTCTAGTGTGGGAG
Nhlh1 -298R	AATAGAGAGCTGGGGAAGGG
Nhlh1 145 F	CCCTTTTGCCTAGGCCTTAC
Nhlh1 145R	CTCAAGGGTCATCTGTCTGC
Nhlh1 314 F	GACAGATGACCCTTGAGCAG
Nhlh1 314R	ATGCTGCCCAAAGAATCCAA
Nhlh1 871 F	ACATGGATGGGAAATGCTCC
Nhlh1 871R	CACCAGCATCTGACACCTAC
Nhlh1 1522 F	GAGGCTGAGGGATTGTGAAG
Nhlh1 1522R	CCTTCTGCACCCTTGTTTCT
Nhlh1 2906 F	TGTCTCGAAGGACTTCACCT
Nhlh1 2906R	AGCCGAACTTGGCTTCATAG
Nhlh1 3683 F	CTCCTACCTGAACCATGTGC
Nhlh1 3683R	AGGGAAATGGGGAGAATCCA
Nhlh2 -4520 F	CGATGCCTCAACACATACCA
Nhlh2 -4520R	TACCCACTCACAGACACACT
Nhlh2 -824 F	TTTGGGACTGGAGGTCATCT
Nhlh2 -824R	CCCATCCTAACACGTGAGTG
Nhlh2 418 F	AGAGGCAACCTTAAGCCCTA
Nhlh2 418R	GGGACGATTCCTCCACTTTC
Nhlh2 2907 F	GAGCTCCGCAAACTACTACC
Nhlh2 2907R	TCAAGTGTCTCTGGGCAAAC
Nhlh2 4798 F	CCAGCCTGGGATGGTATAGA
Nhlh2 4798R	CTCCTTAGCCCCGAGTTTTC
NeuroD1 -1150 F	AACCATTCCTCCTCCTCCTC
NeuroD1 -1150R	CTGTTTCCCTTTCTGGGGAC
NeuroD1 -619 F	AAGGTTGAGTCAAGGCTGTG
NeuroD1 -619R	GGTGGCTGGCTTCTAATCTC
NeuroD1 -338 F	AAGCAGTCTTCAGGCTAGGA
NeuroD1 -338R	ATTAACCCTTTGTGGCAGCA
NeuroD1 328 F	ATTTGTGGAGTCGGTTGTCC
NeuroD1 328R	GAGTGCTGGGACTCATTACG
NeuroD1 830 F	TGCTACCTGTTACTGTCCCA
NeuroD1 830R	GGCTTTTCAAAGTTCGCCTC
NeuroD1 1450 F	GTGAGTTGGGAGTGACTTGG
NeuroD1 1450R	GTCCACTGCAAAATGGATGC
NeuroD1 2258 F	AATAGAGACACTGCGCTTGG
NeuroD1 2258R	CAGGGGACTGGTAGGAGTAG
NeuroD1 2658 F	TGCCTTTACCATGCACTACC
NeuroD1 2658R	GTTGTCTATGGGGATCTCGC
NeuroD1 4271 F	AGCTTGTCCTGTGCTTAGTC
NeuroD1 4271R	TAAAACAGAGGCGAGGTCTG
NeuroD2 -1838 F	GACTTCCTAGTTGCAGAGCC
NeuroD2 -1838R	GAAAGAAGGTCCAAAGGCCA
NeuroD2 -450 F	GGTGCCAGCATCTACCTATG
NeuroD2 -450R	CATTTCCCTGTCTCCAGGTC
NeuroD2 428 F	CCGTGTTCTCTCTCCCATTG
NeuroD2 428R	GTGGGAAAAGGTCACAGGTT
NeuroD2 1388 F	CACTCTGTGCTGTCTGTCTC
NeuroD2 1388R	GGATCTCTTCTCCTCCACGA
NeuroD2 3082 F	CACCCTAACACGAATCTCCG
NeuroD2 3082R	ATGCGTTTTCTCTCCGATCC

## Additional files

Additional file 1: Figure S1. Location of pictures presented and MBD3 expression at E16.5. **(A)** Schematic representation of a sagittal mouse brain showing where rostral, middle and caudal sections were taken. Below are schematics of coronal rostral, middle and caudal sections showing where the displayed pictures were taken. **(B)** Immunostaining for MBD3 on sagittal sections of three WT and three MBD3 cKO brains at E16.5. Scale bar = 100 μm. **(C)** Immunostaining for TuJ1 on posterior coronal sections of WT and MBD3 cKO brains at E12.5, E14.5 and E16.5 taken at low magnification (5×). Additional file 2: Figure S2. No difference in levels of apoptosis detectable in Mbd3 cKO and WT embryonic brain. **(A)** Representative immunostaining of E16.5 brain sections for activated (cleaved) CASPASE-3 (green) alone (left panel) and counterstained with DAPI (blue, right panel). **(B)** Quantification of the mean number of positive cells observed per section at E14.5, 16.5 and E18.5. *N* = 3 to 5, scale bar = 100 μm. Additional file 3: Figure S3. Mbd3cKO embryos have altered cell cycle distribution at E14.5. **(A, B)** Average percentage of PAX6+ cells in each phase of the cell cycle at 16.5 (A, *N* = 2 to 12) and E14.5 (B, N = 5 to 6) **P* < 0.05. Error bars represent st. dev. Additional file 4: Figure S4. No difference in CUX1 staining between WT and MBD3 cKO embryonic brains at E18.5. Representative immunostaining of E18.5 coronal brain sections for CUX1 (green) from WT (left) and MBD3 cKO (right) embryos. Scale bar = 100 μm. Additional file 5: Figure S5. No detectable difference in Cajal-Retzius/layer 1 marginal zone neurons between Mbd3 cKO and wild-type embryonic brain. Representative immunostaining of E16.5 brain sections for Reelin (green) counterstained with DAPI (Blue) from wild-type **(A)** and cKO **(B)** embryos. Additional file 6: Table S1. List of microarray genes in STEM cluster 35. Additional file 7: Table S3. List of microarray genes in STEM cluster 39. Additional file 8: Table S2. GO term analysis of genes in STEM cluster 35. Additional file 9: Table S4. GO term analysis of genes in STEM cluster 39. Additional file 10: Table S5. List of microarray genes in STEM cluster 28. Additional file 11: Table S7. List of microarray genes in STEM cluster 29. Additional file 12: Table S6. GO term analysis of genes in STEM cluster 28.

## Abbreviations

BrdU: bromodeoxyuridine
ChIP: chromatin immunoprecipitation
cKO: conditional knockout
ES: embryonic stem
FDR: false discovery rate
GO: gene ontology
MBD3: methyl binding domain 3
NuRD: nucleosome remodelling and deacetylase
pH3: phosphorylated histone H3
qRTPCR: quantitative reverse transcriptase polymerase chain reaction
STEM: short time-series expression miner
SVZ: sub-ventricular zone
VZ: ventricular zone
WT: wild type
