# Supplementary figures and images for: The methyl binding domain 3/nucleosome remodelling and deacetylase complex regulates neural cell fate determination and terminal differentiation in the cerebral cortex
Source: Neural Dev. 2015 May 2;10:13. doi: 10.1186/s13064-015-0040-z (PMC4432814; doi:10.1186/s13064-015-0040-z)

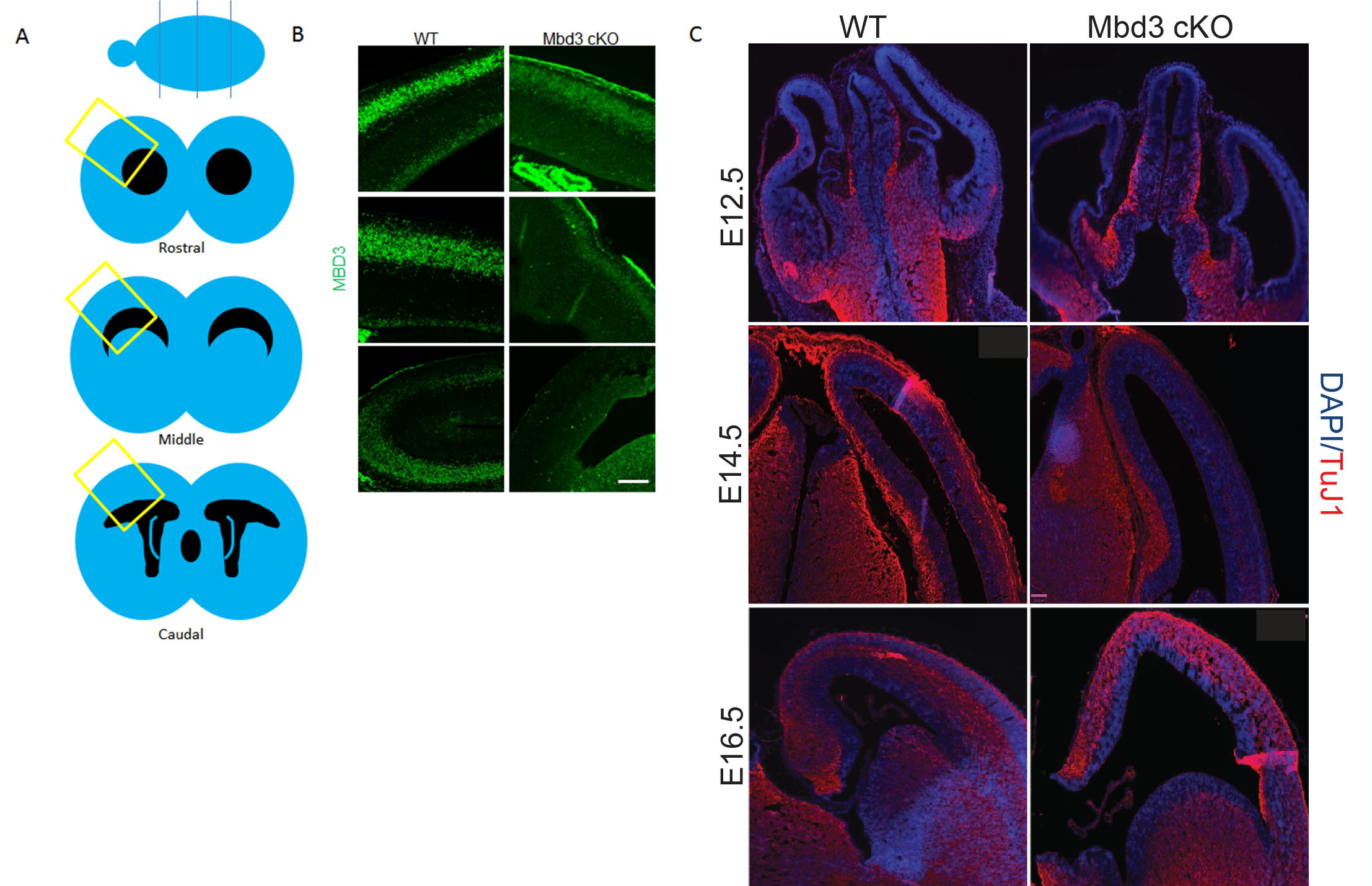

Supplement: Additional file 1: Figure S1. — Location of pictures presented and MBD3 expression at E16.5. (A) Schematic representation of a sagittal mouse brain showing where rostral, middle and caudal sections were taken. Below are schematics of coronal rostral, middle and caudal sections showing where the displayed pictures were taken. (B) Immunostaining for MBD3 on sagittal sections of three WT and three MBD3 cKO brains at E16.5. Scale bar = 100 μm. (C) Immunostaining for TuJ1 on posterior coronal sections of WT and MBD3 cKO brains at E12.5, E14.5 and E16.5 taken at low magnification (5×). [file 13064_2015_40_MOESM1_ESM.jpg]

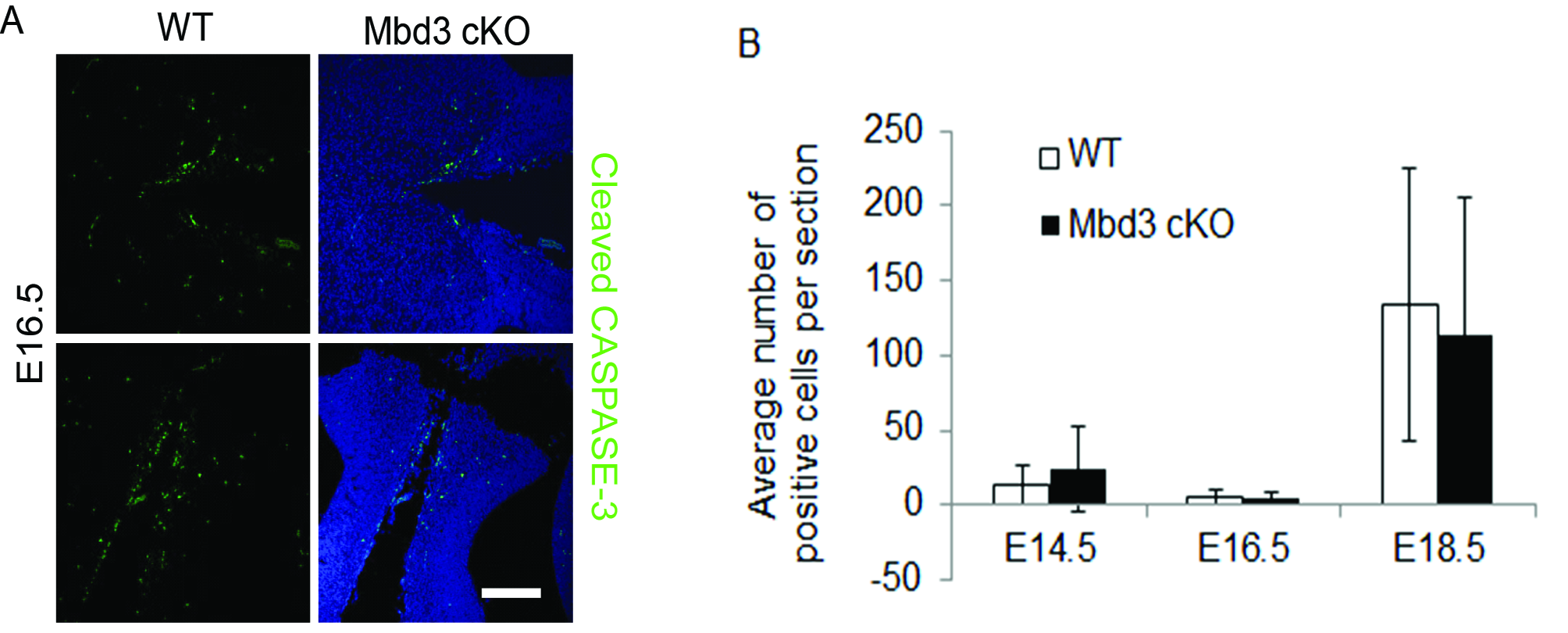

Supplement: Additional file 2: Figure S2. — No difference in levels of apoptosis detectable in Mbd3 cKO and WT embryonic brain. (A) Representative immunostaining of E16.5 brain sections for activated (cleaved) CASPASE-3 (green) alone (left panel) and counterstained with DAPI (blue, right panel). (B) Quantification of the mean number of positive cells observed per section at E14.5, 16.5 and E18.5. N = 3 to 5, scale bar = 100 μm. [file 13064_2015_40_MOESM2_ESM.tiff]

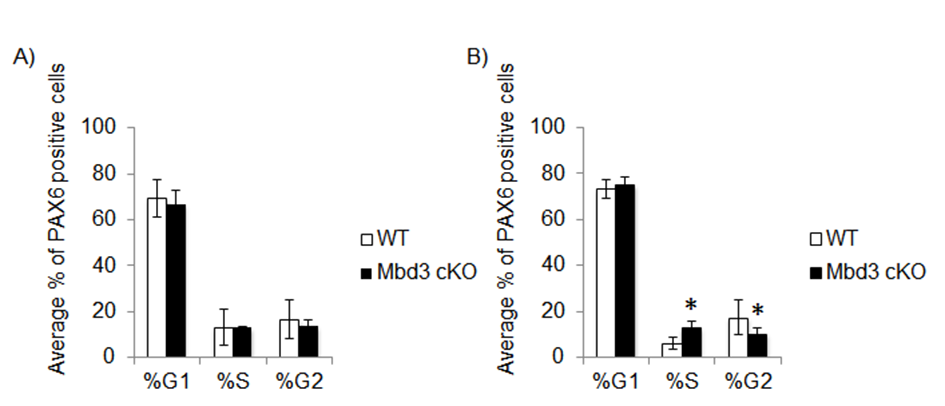

Supplement: Additional file 3: Figure S3. — Mbd3cKO embryos have altered cell cycle distribution at E14.5. (A, B) Average percentage of PAX6+ cells in each phase of the cell cycle at 16.5 (A, N = 2 to 12) and E14.5 (B, N = 5 to 6) *P < 0.05. Error bars represent st. dev. [file 13064_2015_40_MOESM3_ESM.tiff]

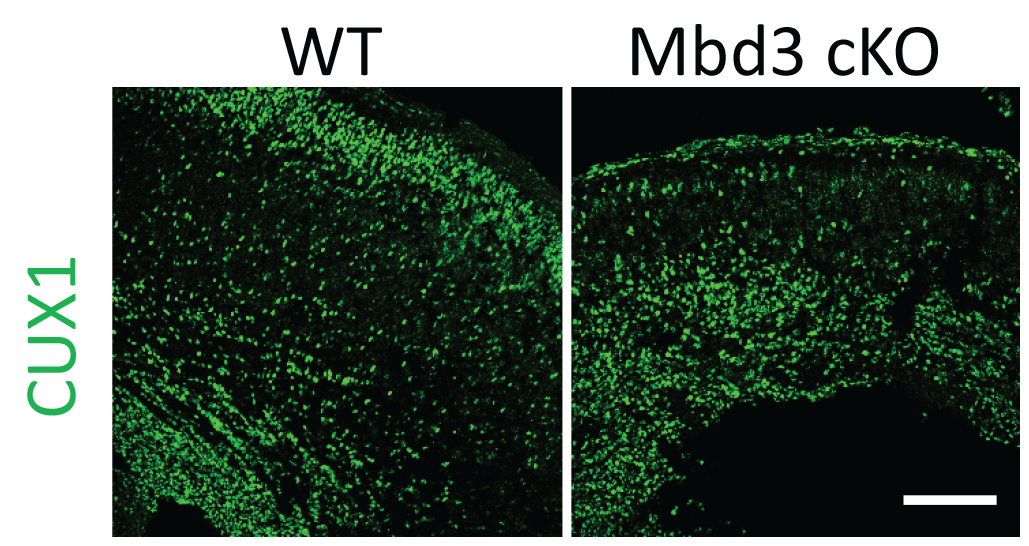

Supplement: Additional file 4: Figure S4. — No difference in CUX1 staining between WT and MBD3 cKO embryonic brains at E18.5. Representative immunostaining of E18.5 coronal brain sections for CUX1 (green) from WT (left) and MBD3 cKO (right) embryos. Scale bar = 100 μm. [file 13064_2015_40_MOESM4_ESM.tiff]

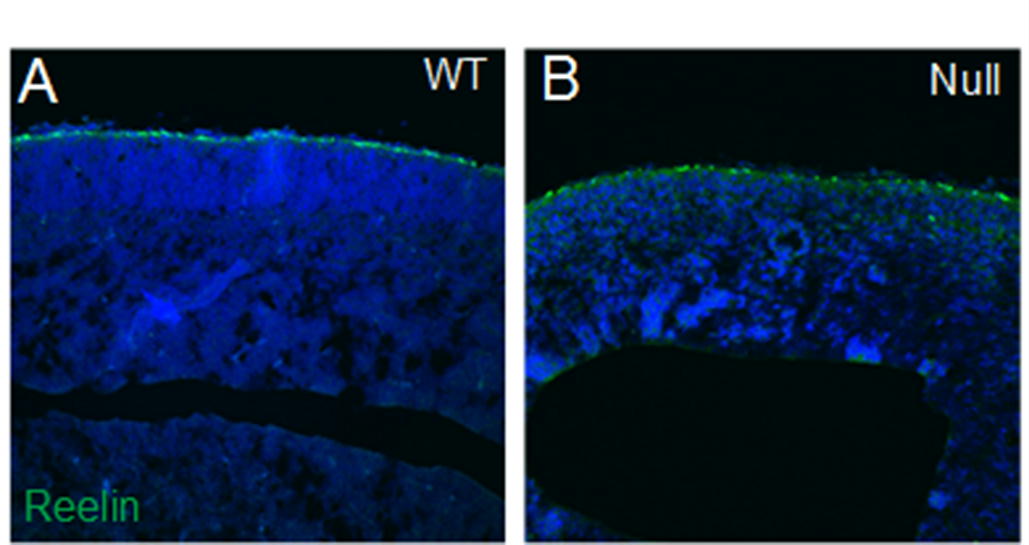

Supplement: Additional file 5: Figure S5. — No detectable difference in Cajal-Retzius/layer 1 marginal zone neurons between Mbd3 cKO and wild-type embryonic brain. Representative immunostaining of E16.5 brain sections for Reelin (green) counterstained with DAPI (Blue) from wild-type (A) and cKO (B) embryos. [file 13064_2015_40_MOESM5_ESM.jpg]
